# All-optical 3D blue phase photonic crystal switch with photosensitive dopants

**DOI:** 10.1038/s41598-024-60667-6

**Published:** 2024-04-30

**Authors:** Eva Oton, Martin Cigl, Przemysław Morawiak, Sergei Mironov, Alexej Bubnov, Wiktor Piecek

**Affiliations:** 1 Faculty of New Technologies and Chemistry, Military University of Technology, Warsaw, Poland; 2 Institute of Physics, Czech Academy of Sciences, Prague, Czech Republic

**Keywords:** Liquid crystals, Photonic crystals, Liquid crystals

## Abstract

Blue phase (BP) liquid crystals (LC) have lately become the focus of extensive research due to their peculiar properties and structure. BPs exhibit a highly organized 3D structure with a lattice period in the hundreds of nm. Owing to such structure, BPs are regarded as 3D photonic crystals. The unique properties of this complex LC phase are achieved by the self-assembly of the LC molecules into periodic cubic structures, producing bright selective Bragg reflections. Novel applications involving 3D photonic crystals would certainly benefit from enhanced ground-breaking functionalities. However, the use of BPs as 3D has been traditionally curtailed by the BP crystals trend to grow as random polycrystals, making it difficult to develop practical BP-based photonic devices. The possibility of generating mm-sized BP monocrystals was recently demonstrated. However, besides increasing the scarce number of 3D photonic structural materials, their applications as 3D photonic crystals do not show apparent advantages over other solid materials or metamaterials. Having a tunable BP monocrystal, where crystals could be switched, modulating simultaneously some of their properties as 3D photonic crystals, they would constitute a new family of materials with superior performance to other existing materials, opening up a plethora of new applications. In this work, an all-optical switchable 3D photonic crystal based on BPs doped with tailored photoactive molecules is demonstrated. Two switching modes have been achieved, one where the BP reversibly transitions between two BP phases, BPI and BPII, (two different cubic crystal systems) while maintaining the monocrystallinity of the whole system. The second mode, again reversible, switches between BPI and isotropic state. None of these modes are related to the regular thermal transitions between LC phases; switching is triggered by light pulses of different wavelengths. This all-optical approach allows for a seamless fast remotely controlled optical switch between two 3D photonic crystals in different cubic crystal systems and between a photonic crystal and an isotropic matrix. Applications of switchable BPs for adaptive optics systems or photonic integrated circuits would make great advances using 3D photonic crystal switches. All-optical photonic systems such as these hold great promise for the development of tunable and efficient photonic devices such as dynamic optical filters and sensors, as they enable light-driven modulation and sensing applications with unprecedented versatility.

## Introduction

In recent years, blue phase (BP) liquid crystals (LCs) have emerged as a promising area of research within the field of photonics, due to their unique properties and structure. Blue phases appear in chiral liquid crystal phases, where the molecular arrangement is driven by the high twisting of the director^1^. Unlike other LC phases, BPs exhibit a highly organized 3D structure with a lattice period in the order of hundreds of nanometers. This structure is formed by the self-assembly of LC molecules into periodic cubic structures that produce bright selective Bragg reflection in narrow bands. With their peculiar structure and properties, BPs are considered 3D photonic crystals^1,2^. BPs sub-micrometer cubic structure is optically isotropic by symmetry. Unlike cholesteric liquid crystals, which only present Bragg reflection in one dimension, BPs present periodicity in all three dimensions, and as a result, produce multiple reflections in different directions^3^. Furthermore, BPs offer electric field-driven switching with sub-millisecond response times, making them attractive for applications requiring rapid modulation and switching of optical signals^4,5^.

Previous research focused on the engineering of the properties of BP crystals has successfully shown that new functionalities can be induced into the BP structures^6–9^, and various novel photonic applications will surely benefit from the additional versatility^10–13^. Adding different dopants to the BPs can produce enhanced or even new optical properties from such doped structures^14,15^.

A current exciting avenue relies on providing BP crystals with photoswitching properties induced by light stimuli alone^16^. Photoresponsiveness and photomodulation have been studied by introducing photosensitive materials into BP structures^17,18^. Phase induction and bandgap change upon exposure to UV light have been demonstrated^19–21^.

However, the main problem faced by all studies is that direct applications for BPs are often hindered by the crystals’ tendency to grow as disorganized polycrystals^22^. Polycrystalline structures—known as platelet structures—are made of many small, disorganized BP crystals. The colorful appearance of the platelet texture is the result of the Bragg reflection from particular lattice planes because each platelet is a cluster of several BP cubes in one specific lattice orientation. Polycrystalline BPs, though, tend to produce scattering and, as a result, poor optical and electrooptical properties^23^.

The effective control of BP alignment is an actual issue that needs to be addressed if this innovative material is targeted to be implemented in novel photonic applications. Creating large BP monocrystals is however a non-trivial task that becomes more complicated when the control of the lattice orientation is involved. BPs have been previously produced in virtually ideal small crystals by various methods, for instance, by orienting layers, by a long-lasting applied voltage, microwell confinement, or photopatterning^24–27^.

In our previous research^28^ we demonstrated that by imposing specific smart surface anchoring properties, large monocrystalline BPs could be obtained. Using designed precursor mixtures, a crystal with a chosen spatial orientation of the lattice can be obtained as well. The BP crystals were grown in different unique lattice orientations, in millimeter-sized crystals, and with excellent optical properties. The control of both lattice plane and azimuthal orientation results in one particular 3D orientation, which is driven by the anchoring energy, where the interfacial free energy induces some lattice orientations and prevents others^29^.

In this work we have introduced novel tailored photoactive dopants into the BP monocrystal structure, obtaining a large phototunable 3D photonic crystal system at chosen lattice orientations. Custom-designed photoactive dopants are based on azobenzene and the large shape change accompanying the photochemical *E–Z* isomerization works like a molecular switch, triggering macroscopic changes in the structure through the cooperative effect of the BP host molecules^30^. Photoactive dopants (PDs) can influence and tune the stability, structure, and photonic bandgap of BP phases^31,32^. This strategic addition aims to harness the photoswitching capabilities of doped BPs under external light stimuli.

We demonstrate an all-optical switch of the BP macroscopic monocrystal, where a sizeable blue phase I (BPI) monocrystal can rapidly transition into a blue phase II (BPII) monocrystal, and by irradiating at a different wavelength, the BPII can swiftly transition back to BPI maintaining the monocrystallinity of the BP phases. In addition, using different BP precursor mixtures, it was possible to inhibit the appearance of BPII, thus obtaining a photoswitchable BPI monocrystal shutter, transitioning between monocrystalline BPI and isotropic state, in a reversible manner as well., Besides, we selectively patterned a BPI crystal with a BPII stripe, by exposing a BPI with a straight-line pattern, effectively changing the exposed areas to BPII.

We thus confirm that it is possible to seamlessly switch between BP phases with optical means only while preserving the monocrystalline structure of the BPs in all cases by using specific surface and precursor mixture conditions.

The possibility of dynamic control of 3D Blue Phases is a highly interesting research line to explore for photonic applications. Light possesses peculiar benefits like wireless guiding, multidimensional controllability, and precision among other benefits. Incorporating azobenzene PDs into BPs can be valuable in various applications^33,34^. The range of potential applications includes light-driven displays with dynamic and programmable features, tunable optical filters for telecommunications, and advanced sensors with responsive optical characteristics. The ability to precisely control the optical properties of BPs through photoswitching may find applications in adaptive optics, where rapid adjustments in optical elements are crucial for optimal performance in imaging systems, astronomical instruments, and laser systems.

## Results

### Photoresponsive BP monocrystals

The introduction of photosensitivity to BP crystals is usually realized by doping a BP host with azobenzene derivatives. After some initial tests, four new materials with very stable azobenzene photoactive units were designed to closely meet the requirements of BP crystals, specifically the speed, effectivity, stability of photoinduced changes, and their compatibility in the LC matrix.

Two different illumination sources were needed for inducing the reversible photoisomerization of PDs: a 365 nm LED spot lamp and a 550 nm LED source with intensity regulation for isomerization control: when irradiating the PD doped BP crystals at 365 nm, the dopants undergo an *E–Z* photoisomerization, and this molecular change produces a wide-range change in the crystalline structure of the BP monocrystals. Subsequent illumination with the 550 nm light source provides a *Z–E* isomerization reversing the switching process.

Interestingly, we found that the dopant *E–Z* isomerization induces a quick phase transition in the BP monocrystals. Using the same methods as in^28^, three BP precursor mixtures, BP#1, BP#2, and BP#3 were prepared, all containing the same nematic base but a different concentration of a chiral dopant for each mixture. Each precursor mixture was then divided and used to prepare four mixtures, each one doped with one of the four PDs (MCF526, MCF528, MCF613, and MCF619) at 1 wt%. Each mixture was then filled into glass cells.

Mixture *BP1#1* was formulated to induce a monocrystalline BPI in the lattice orientation (110). The mixture was filled into glass cells (size ca. 1.5 × 1 cm, 10 μm cell gap) with coated surfaces in isotropic phase and then cooled down at 0.5 °C/min temperature rate. The phase sequence from isotropic (ISO) to cholesteric phase (CLC) in this precursor mixture was determined as: ISO–52.7–BPII (100)–51.4–BPI (110)–49.3–CLC. For the BP phases, both BPII (100) and BPI (100) covered the full cell area as monocrystals at their respective temperature ranges—verified by polarizing optical microscopy (POM) texture analysis, Kossel diagram, and spectral analysis.

### All-optical blue phase monocrystal switch

**Figure 1:**
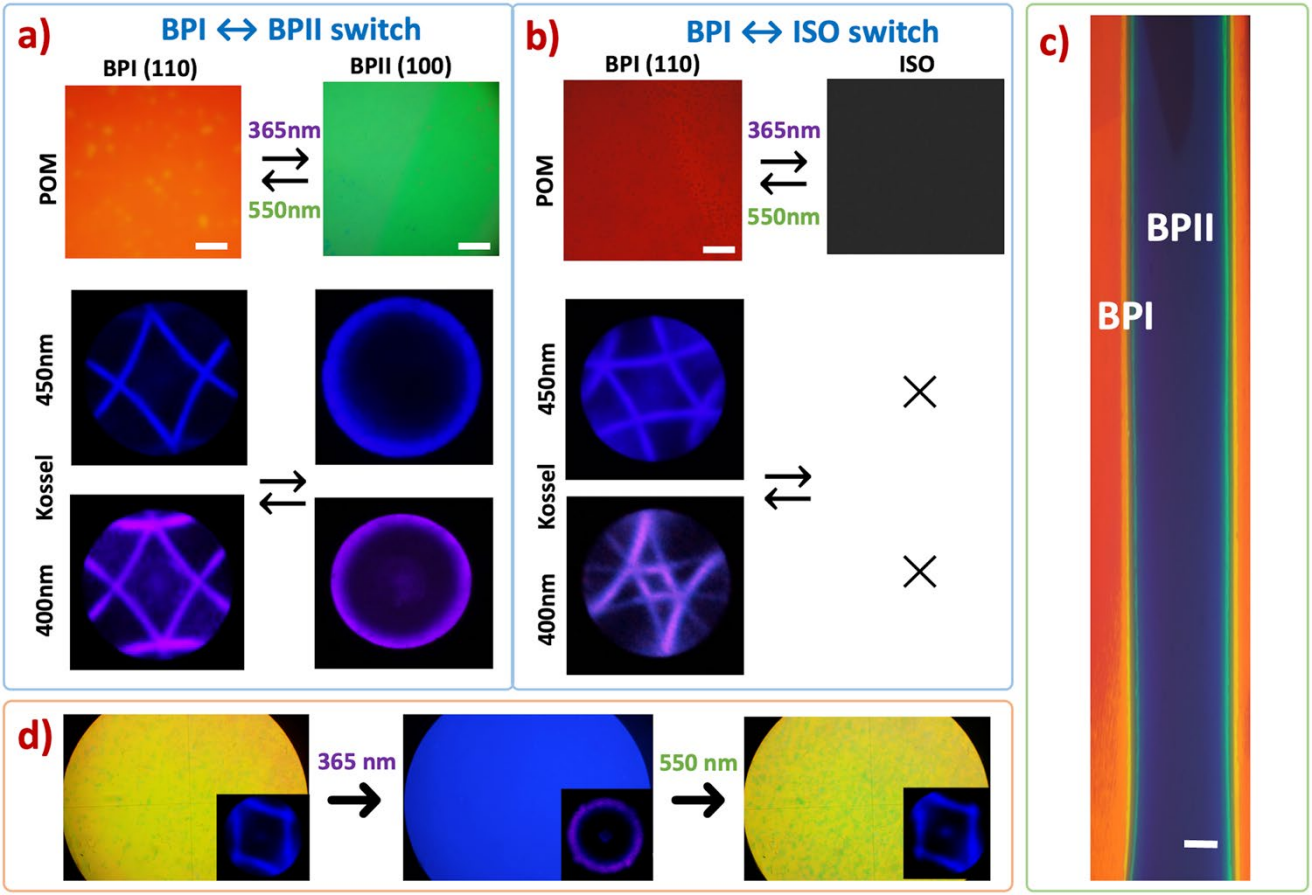
Reversible photoswitching behavior of BP monocrystals: (**a**) switch between BPI (110) and BPII (100) (BPI#1), (**b**) switch between BPI (110) and isotropic (BP#2), (**c**) selective BPII stripe pattern in a BPI: BPI (110) (red) BPII (100) (blue), (**d**) photoswitching illumination sequence on a BPI⟷ BPII switch doped with photosensitive dopant MCF613 (BP#3). All BP photoswitching processes are reversible and structures remain monocrystalline. All white scale bars correspond to 1.0 mm.

Figure 1a shows a BP monocrystal switch upon irradiation with either a UV or green light source. For each of the four types of PD doped cells, a BPI (110) was formed from the isotropic state at their respective temperatures, which varied slightly depending on the type of PD used in the precursor mixture. Instantly after UV (365 nm) illumination, the BPI (110) monocrystal transformed into BPII (100), producing a uniform texture along the whole illuminated area as seen by POM and Kossel analysis. The photogenerated BPII (100) state—that is, the BP matrix with the majority of PDs’ molecules in the form of *Z* isomer—is stable and remains in this state as long as it is not illuminated with the green LED light source, λ = 550 nm, (checked for 6.0 + h). When switching to the green light source, the BPII (100) transforms back to monocrystalline BPI (110).

A second BP photoswitching system was designed, where the BPI monocrystal becomes a selective shutter. It demonstrated reversible switching transitioning between BPI and isotropic state. BP precursor mixture, *BP#2*, was prepared with a different concentration of the chiral dopants, in a range where the appearance of BPII can be inhibited. The phase transition temperature of this precursor mixture was: ISO–53.3–BPI (110)–48.9–CLC. In fact, the BPII phase is not completely inhibited, but the temperature range where it appears is negligible for our purposes in this work (ΔT_BPII_ < 0.2 K).

Like before, four mixtures were prepared from this precursor with the four types of PDs and filled into glass cells. The BPI to isotropic (BPI⟷ ISO) switch is shown in Fig. 1b. Again, for each PD doped sample, the BPI (110) was induced from the isotropic state. After UV illumination, the BPI (100) monocrystal transitions into isotropic state and reverses back to BPI (110) when the system is switched using the green light source.

We tested the remarkable selectivity of this system by successfully patterning a PD doped BP in a stripe design. A BPI (110) monocrystal was induced upon cooling from the isotropic state, covering the whole active area of the glass cell (1.5 × 1.0 cm). The BPI (110) was then illuminated with the UV source in a straight-line pattern with an effective illumination spot of *d* = 1.5 mm in diameter, *P* = 4 mW/cm^2^ and exposure speed, *v* = 1.5 cm/s, which accurately changed the exposed areas to BPII (100). As shown in Fig. 1c, the selective BPI/BPII pattern shows a blue stripe that corresponds to BPII (100), while the red stripes show the unexposed BPI (110). An exposure of the whole cell area with λ = 550 nm erases the full pattern switching all the BPII back to BPI (110). Smart writable/erasable patterns can be generated using this system. Reconfigurable BP photonic crystals able to be repeatedly recorded could be employed in diverse photonics applications such as adaptive holographic systems.

By illuminating the BP crystals back and forth, the all-optical BPI⟷ BPII and BPI⟷ ISO switches can be continuously written and erased. This is illustrated in Fig. 1d, where a BP crystal was sequentially switched: BPI (110) was irradiated at 365 nm switching to BPII (100) and then back to BPI (110) at 550 nm. The sequence was repeated in a cycle; the third POM image shows the BPI (110) monocrystal texture and Kossel diagram after 25 cycles (Fig. 1d).

The total number of cycles (at sufficiently high illumination levels) does not seem to play such a critical role in the efficiency of the BP crystal switch. After 80 cycles of UV/green irradiation, the BP phases still remained as well organized monocrystals, whose Kossel diagrams, reflection spectra, and texture uniformity did not significantly vary over each illumination cycle. This repetitive cycle was performed on mixture *BP#3* doped with one of the PDs which bore slightly higher concentrations of the chiral dopants, hence the BP phases bandgaps are blue-shifted compared to *BP#1* and *BP#2*.

Having a BPI⟷ BPII fast transition implies a switch between two 3D photonic crystals in different cubic crystal systems (BCC and SC respectively). This dynamic transition between crystal structures could hold significant potential for highly tunable and efficient photonic devices, like adaptive optics systems or photonic integrated circuits. Additionally, the fact that both BPs display very different reflection bands could be beneficial in wavelength-selective applications, like dynamic optical filters and sensors.

Moreover, a BPI⟷ ISO optical switch expands the capabilities of this system by acting as a selective light shutter, between the reflective BPI and isotropic state, which could be used in a number of additional photonic devices, by precisely manipulating the light exposure, as in optical networks to route and switch signals efficiently or optical security devices.

### Photosensitive dopants design

**Figure 2:**
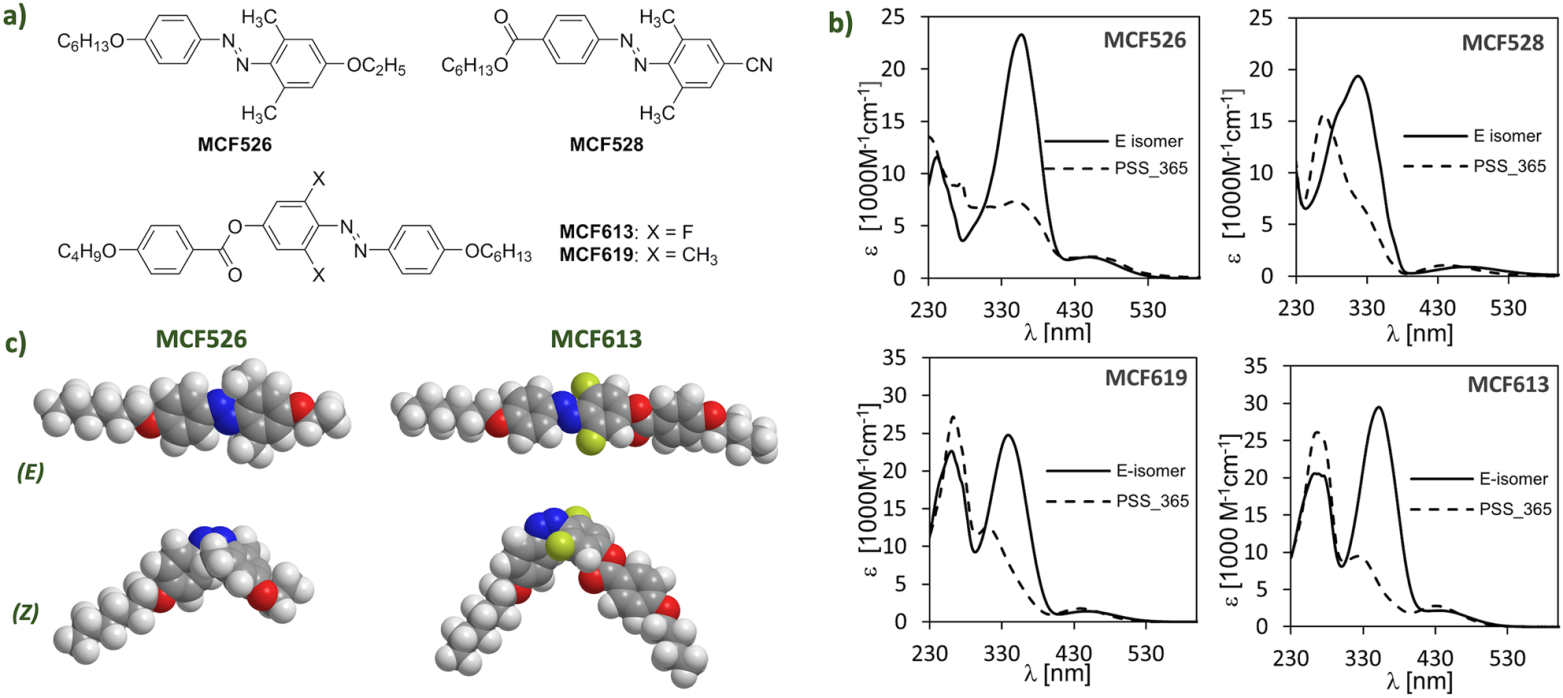
The four photoactive dopants used in this study: (**a**) molecular structures, (**b**) UV–vis absorption spectra, and (**c**) molecular visualizations of E and Z isomers for MCF526 and MCF613 PDs.

In order to pursue better switching properties and compatibility with the BP matrix, compared to commercial azobenzene compounds, we designed and synthesized four new PDs based on azobenzene, including non-mesogenic and mesogenic compounds (see Supplementary Information (SI) for the synthetic procedures and mesomorphic behavior). The molecular structures of dopants are shown in Fig. 2a. Non-mesogenic dopants MCF526 and MCF528 are electron rich and electron poor azobenzene derivatives, respectively. This is given by their substitution by an electron donating alkoxy substituent or an electron withdrawing carboxy or cyano group placed on benzene rings in the *para* positions to the azo bond. Electron rich azobenzenes are the most frequently used PDs, with a high absorption in the UV-A region, thus resulting in a high photosensitivity^35^. In contrast, electron poor azobenzenes do not show such high absorption and photosensitivity, which, in principle, offer an easier adjustment of photoinduced effects simply by changing the irradiation time. However, the synthesis of such materials is challenging compared to electron rich azobenzenes. The mesogenic nature of PDs MCF613 and MCF619 was expected to ensure a better correlation with the LC matrix resulting in a faster response to light stimuli and much stronger photoinduced changes. Increased molecular length of mesogenic PDs also provides a higher absolute molecular shape change upon isomerization, which can provide a stronger photoinduced effect. Moreover, all designed materials possess small bilateral substituents to stabilize their photogenerated *Z* isomers and thus stabilize photoinduced changes. As has been already shown before, bilateral substitution is a useful tool in tuning the kinetic stability of photoinduced changes of azobenzene-based photosensitive LCs, the UV–Vis spectra characteristics and it can also improve the compatibility of PDs with the LC matrix^36,37^.

Before use in the BP matrix, we tested the *E–Z* photoisomerization behavior of pure PDs under UV light (365 nm) in solution. PDs based on electron rich azobenzene (MCF526, MCF613, and MCF619) showed ca. 95% of *Z* isomer in the photostationary state (PSS) while the electron poor azobenzene derivative MCF528 showed only 30% and thereby was expected to be less effective in photoswitching. The two mesogenic compounds MCF613 and MCF619 excelled in the time necessary to reach PSS. It only took several minutes to reach PSS in comparison to non-mesogenic DPs, where ca. 20 min of irradiation was required. These observations can be partially rationalized if we compare the UV–Vis absorption spectra of PDs (Fig. 2b, see also Table S2 in Supplementary Information (SI) for further details).

Mesogenic materials show higher differences between the absorption spectrum of pure *E* isomer and *Z* isomer rich PSS, which is the prerequisite of the high content of photogenerated isomer at the used irradiation wavelength (Table S3 in SI). The low content of *Z* isomer in the PSS mixture found for MCF526, which also showed the lowest absorption at 365 nm, is in line with this argument. Nevertheless, we realize that the PSS composition also depends on quantum yields of *E–Z* and *Z–E* photoisomerization processes, which were not determined in our study, and the thermal *Z–E* isomerization process, which is negligible in our case. The overall effectivity of the *E–Z* photoisomerization process at the used wavelength could be the reason why MCF526 showed 95% of *Z* isomer in PSS, despite having similarly low differences in the absorption spectrum at 365 nm as MCF528.

The kinetic stability of the photogenerated *Z* isomer was measured by proton NMR spectroscopy directly in PSS mixtures in deuteriochloroform. The observed thermal *Z–E* isomerization was less than ten percentage points per day for all materials at 20 °C, which is a very good stability for such simple molecular structures. Since the rate of the thermal *Z–E* isomerization depends on the polarity of the medium^38^ and a few other parameters in ordered media like liquid crystals^39^, these data only served as a qualified guess for us.

### BP photoswitch phase characterization

In order to study the phase sequence of the BP mixtures under the influence of photoisomerization, all samples were cooled down from isotropic state, first without inducing the *E–Z* photoisomerization. Naturally, to observe the phase transitions and corroborate the BP lattice orientation by Kossel analysis, the samples must not be illuminated with wavelengths below 500 nm to prevent isomerization. For that reason, the whole phase sequence before inducing isomerization was performed at 550 nm, measuring the Kossel diagram sequence while cooling. Next, the samples were heated to isotropic state again and illuminated with a lamp spot at 365 nm for 30 s. The phase transitions were then recorded while measuring the Kossel diagram transition with a 400 nm microscope light source.

**Figure 3:**
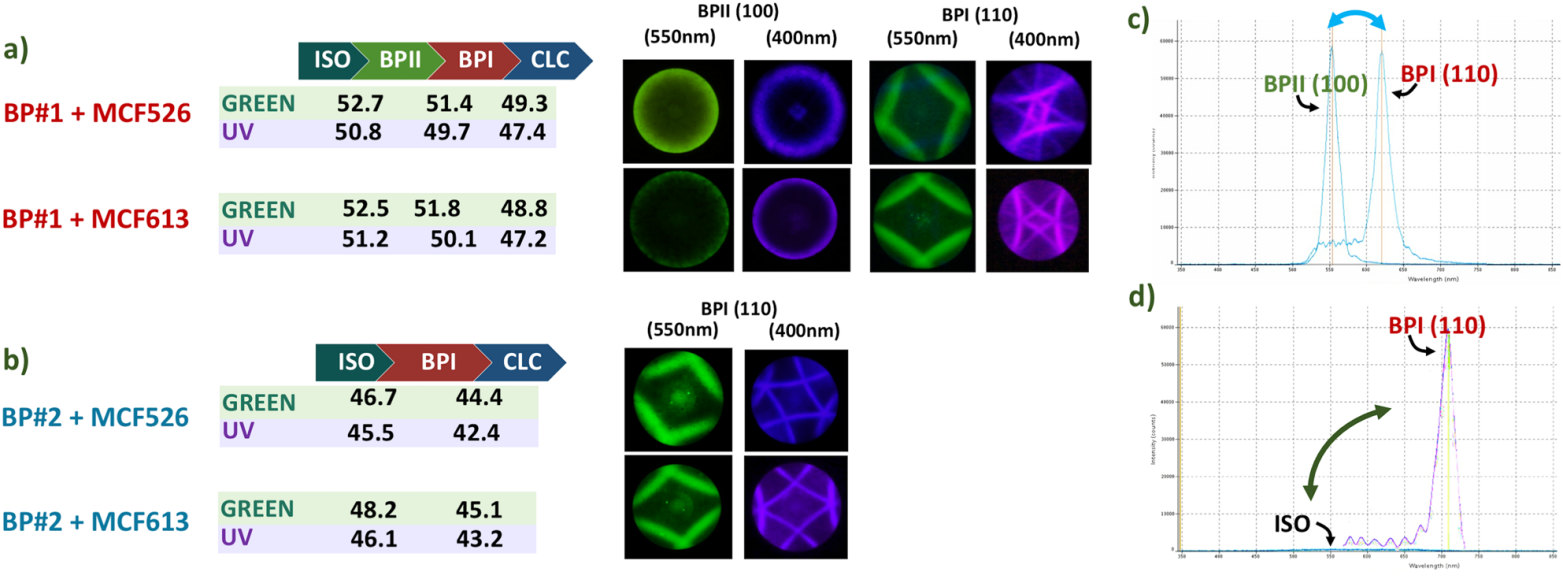
BP transition temperatures, Kossel and spectra analysis: (**a**,**b**) transition temperatures (in ºC) of mixtures BP#1 and BP#2 doped with either MCF526 or MCF613 and while being illuminated with a green or UV light source. (**c**,**d**) Reflection spectra of the BPI⟷ BPII and BPI⟷ ISO optical switches.

Phase transition temperatures were measured for mixtures BPI#1 and BP#2 doped with all four PDs. For BPs doped with MCF526, MCF613, and MCF619, all phase transition temperatures shifted to lower temperatures when the samples were under UV instead of green illumination. For BPs doped with MCF528, phase transition temperatures did not show a noticeable shift. Even when the illumination power was increased twice, there was no visible phase or texture color variation in the BP phases either. As shown above, a maximum of 30% of MCF528 molecules are converted to *Z* isomer and, at the concentration level used in the BP mixture, this is not enough to trigger any changes, even though the BP is a very sensitive mesophase. Figure 3a, b show the phase transition temperatures for mixtures BPI#1 and BP#2 doped with MCF526 or MCF613 with either green or UV illumination. Transition temperatures and optical responses were almost identical for MCF613 and MCF619. All phase transition temperatures shifted to lower temperatures—up to ∼ 2 K difference in some cases—thus allowing a temperature range shift wide enough for the BP phase to switch. Naturally, as per the standard behavior of BP phases the BP lattice size underwent a slight expansion or contraction for BPI and BPII during cooling. The Kossel pattern evolution was recorded while cooling down from isotropic phase with green or UV illumination to verify all the phase transition temperatures.

Spectral measurements were performed on all BP phases: in Fig. 3c the reflection band jump is shown for BPI#1 doped with MCF526. The BPI⟷ BPII switch shows a change from BPI (110), λ = 615 nm switching to BPII (100), λ = 553 nm and back. Figure 3d shows the BPI⟷ ISO shutter reflection bands, switching between BPI (110), λ = 712 nm, and isotropic (no reflection) for mixture BPI#2 doped with MCF526. All BPI (110) and BPII (100) phases covered the full cell area as monocrystals at their respective temperature ranges—verified by POM texture analysis, Kossel diagram, and spectral analysis.

All BP lattice sizes were estimated through Kossel analysis and spectral analysis. From the experimental reflection spectra, lattice sizes were calculated by the BPs reflection band wavelength values, *λ*_*hkl*_, which are given by $\lambda =\frac{2na}{\sqrt{{h}^{2}+{k}^{2}+{l}^{2}}}$, where *a* is the lattice constant, *n* is the average refractive index of the BP matrix assumed to be n ≈ 1.6, and the lattice orientation of a crystal plane is given by the Miller indices (*hkl*).

**Figure 4:**
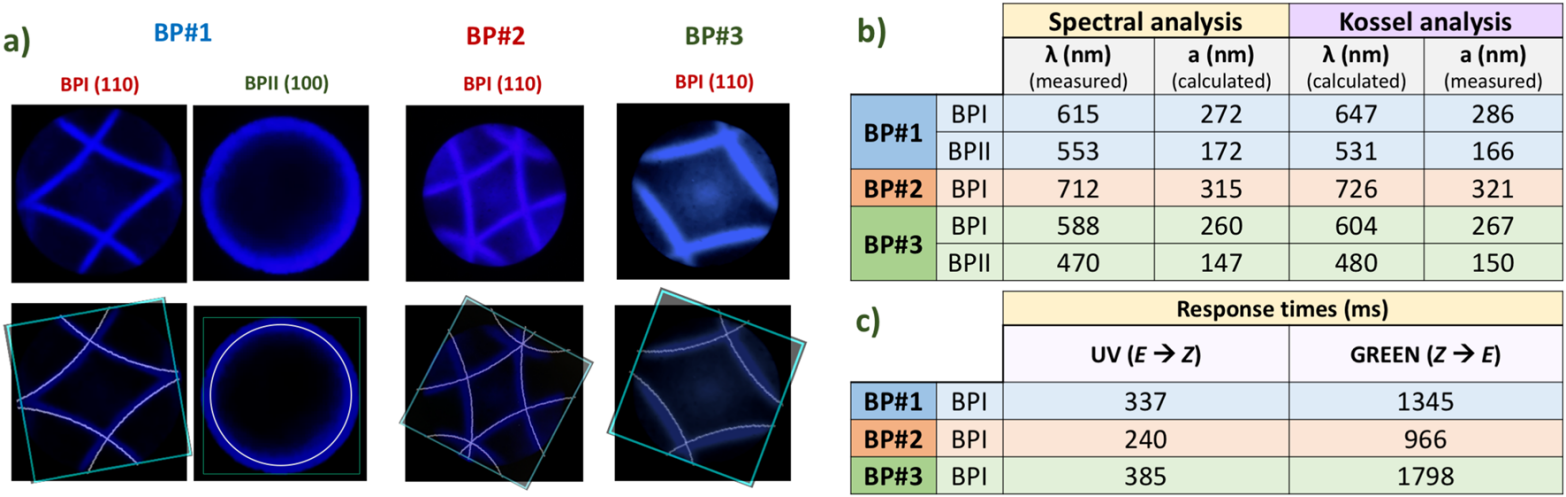
(**a**) Measured and calculated values of the lattice constant and reflection wavelength by Kossel analysis or spectral analysis. (**b**) Kossel pattern fitting: orthographic projections of the Kossel lines fitting the experimental results to estimate the lattice size. (**c**) Response times.

Kossel analysis was accomplished by modeling a body-centered cubic (BCC) or simple cubic (SC) crystal. The estimated lattice constants were obtained with orthographic projections of the Kossel lines fitted to the experimental results as depicted in Fig. 4a, where simulations of the resulting Kossel lines were adapted for converging monochromatic light sources of 450 and 400 nm, and the viewing area from the numerical aperture of used microscope objective. Theoretical reflection bands values were estimated from the lattice size fitting.

Reflection bands measurements and estimations by means of spectral or Kossel analysis are summarized in Fig. 4b, showing a good correlation between experimental observations and calculated values. The average response times for the BP phase switches are summarized in the table in Fig. 4c. The processes driven by UV light are much faster than the recovery of the initial BPI phase by using green illumination at 550 nm. This is caused by a very low absorption of the PDs at 550 nm (see Fig. 2b), which is ca. three orders of magnitude lower than the absorption at 365 nm (see Table S2 in the SI). The response times appeared, in general, slightly faster for the BPI-ISO transitions.

## Discussion

A BP optical switch has been accomplished by doping tailored azobenzene photosensitive dopants successfully inducing a phase change by light stimuli only. Both BP phase changes from BPI to BPII and from BPI to isotropic phase are reversible and maintain their monocrystalline nature during the cyclic photoswitching process.

It is important to mention, though, that this phase switch is by no means the standard phase change caused by temperature variations. First, the phase changes induced by light stimuli happen very quick, as opposed to standard phase transitions when cooling or heating BP phases which can take at the very least several tens of seconds or even a few minutes. Moreover, we also took into account that the heat of the light source could somehow impact sample observation; however, the selected illumination power was in fact insufficient to induce any phase change into the BP crystals. This was verified by illuminating a BPI (110) and a BPII (100) in a mixture without any dopants at the same irradiation powers—no texture, color or phase changes were observed, even when the illumination power was increased to three-times the power that was used for the actual measurements.

Indeed, phase transition temperature shifts are in accordance with an *isothermal phase transition* mechanism. Before UV illumination, all PD molecules are in the rod-shape like *E* isomers, compatible with the LC matrix, thus these molecules can easily get aligned along the highly twisted configuration of the director in the BP phase. After UV illumination, the *Z* isomers voluminous nonplanar bent-shaped molecular configuration increases the steric bulk in the BP matrix, as depicted in Fig. 2c, distorting the structure and reducing the order parameter, which, in turn, reduces the phase transition temperatures of the system.

Given by the non-zero absorption of both isomers at used wavelengths, *E*–*Z* and *Z*–*E*, photoisomerization processes are in a dynamic balance and the system is never completely in *Z* or *E* configuration. Such dynamic process dissipating the thermal energy to the system could certainly be a cause for misalignments or lower order parameter of the BP crystals. The loss of monocrystallinity can be estimated by changes in the sharpness of the Kossel lines. Blurry Kossel patterns indicate there is a loss of monocrystallinity in the bulk because the resulting diffracted patterns are an average of the collective lattice orientation changes^40^. As expected, the illumination power played a considerable role in the speed that the optical BP switch worked. All samples that were irradiated at 4 mW/cm^2^ showed complete phase change switching (BPI⟷ BPII or BPI⟷ ISO) with the dopants, MCF526, MCF613 and MCF619, which possess a high photosensitivity. Consecutive lower irradiation levels produced slower phase switching times up to 0.4 mW/cm^2^ where the BP crystals start showing a loss of monocrystallinity, as observed by increasingly blurry Kossel lines, non-uniform BP textures and the appearance of new reflection bands in the reflectivity spectra measurements. Naturally, the phase transitions are not complete at very low irradiation levels and a coexistence of BP phases was present at this point.

The BPII and BPI phase transition and lattice orientation relationship was studied, as well. We know from our previous works^28,41^ that certain BPI orientations are obtained from particular BPII orientations. A given BPI lattice orientation is preceded by one of the two following paths starting at a particular BPII. A BPII (100) becomes either BPI (200) or BPI (110). For BPII (110), the obtained BPI is either BPI (110) or BPI (211). Specific phase transitions are related by the close proximity of the symmetry between particular lattice orientations and an equivalence between distinct crystal axes, hence specific pairs of BPII and BPI lattice orientations are prone to appear together. In our case, BPII (100) is closer by symmetry to BPI (110) and to BPI (200) than to other lattice orientations; the energy barrier required for rearranging a BPII (100) crystal would be higher if it were to rotate to BPI (211) for instance. For this work all BP precursor mixtures were specifically formulated to avoid the appearance of BPI (200) which can sometimes coexist with BPI (110) and would then hinder the appearance of a single BPI (110) monocrystal.

Controlling additional BP crystal orientation switches would surely be beneficial for smart advanced applications, with high tunability of the bandgap and rearrangement to different lattice orientations. This additional tunability is out of the scope of the present paper; however, further research on this subject is in progress at the moment.

## Conclusions

An all-optical switchable 3D photonic crystal was demonstrated by doping monocrystalline BPs with newly synthesized photosensitive derivatives. Two systems were designed: first, a photoswitch between BPI (110) and BPII (100), which can change back and forth the two BP phases only by changing the light source and without loss of monocrystallinity of the BP crystals. The second is a light shutter, switching between the reflective BPI and the isotropic state, again maintaining the monocrystallinity, and ensuring there is a precise control over the material’s optical properties and the system light conditions. Energy-efficient, all-optical elements based on phototuning of BPs could be pursued by designing on demand PDs compatible with BPs. New design of photosensitive dopants proved successful in obtaining highly effective photosensitive system with great thermal stability. We intend to exploit this design and synthesize chiral and reactive PDs for advanced polymer-stabilized and polymeric BP systems. This work provides insight into driving the application of BPs to tangible photonic devices, aiming to contribute to the evolving understanding of these unusual materials, and in particular, boosting their functionalities for an efficient use in innovative photonic applications.

## Methods

### Synthesis of PDs

**Figure 5:**
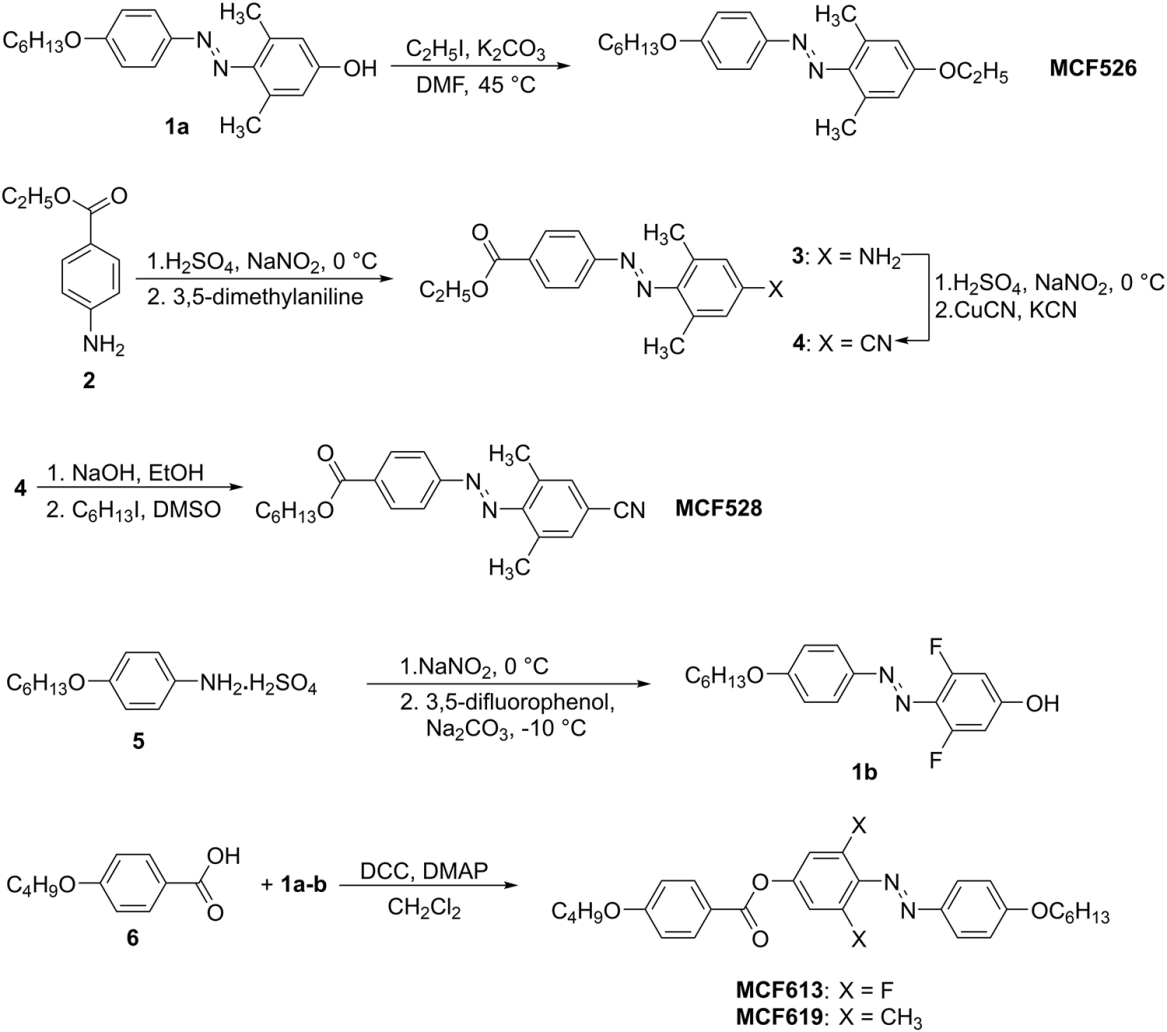
Synthetic pathway for the designed PDs.

Photosensitive dopants were synthesized as shown in Fig. 5. Electron rich non-mesogenic azobenzene dopant MCF526 was prepared by alkylation of the azobenzene derivative **1a**, which was synthesized as we reported in^42^. Since the electron poor azobenzene compounds cannot be synthesized by a direct azo-coupling reaction, had to develop a completely new synthetic strategy.

First the diazotization of aminobenzoate **2** and the azocopulation of the formed diazonium salt with 3,5-dimethylaniline under slightly acidic conditions yielded aminoazobenzene **3**, which was converted to the cyano-intermediate **4** by another diazotization followed by Sandmeyer reaction with copper cyanide. Synthesis of the fluorinated azobenzene derivative started from alkoxyanilinium salt **5**, synthesized as described in^42^. Diazotization and azocopulation reaction with 3,5-difluorophenol under basic condition and low temperature yielded key azophenol **1b**. mesogenic PDs were synthesized from azophenols **1a-b** via DCC-mediated esterification reaction with 4-butoxybenzoic acid (**6**). For detailed synthetic procedures and chemical characterization see SI.

### Isomerization study

UV–Vis spectroscopy was used for the photoisomerization study. A solution of PD in 1,2-dichloroethane in a quartz cuvette of optical length 1 cm was irradiated with UV-LED (365 nm, 140 mW/cm^2^) and the gradual changes we recorded using a spectrometer Schimadzu UV-2600 until there were no further changes upon continued irradiation detected, i.e. the photostationary state was reached. The concentration of the sample was 15 mg/l, which is in the region where Beer–Lambert Law is valid.

The kinetic stability of *Z* isomers of PDs was investigated using a Varian VNMRS 300 spectrometer. The deuteriochloroform solution of PDs in an NMR cuvette was degassed by sonication and flushed with nitrogen to avoid the decomposition of chloroform under UV in the presence of oxygen. Photostationary state mixtures were obtained by irradiation of the deuteriochloroform solutions in NMR cuvettes using the same irradiation source as for the UV–Vis study. Irradiated samples were kept in the non-transparent container submerged in the thermostatic bath (20 °C) and all the handling of the samples was performed in the dark. The changes of integral intensities of several well-separated signals of *E* and *Z* isomer in the proton spectrum were periodically monitored.

### BP mixture preparation

BP precursors contained an in-house formulated mixture whose major compositions are fluorinated terphenyls, biphenyls, and cyclohexylbiphenyls as a nematic host mixture and the chiral dopant ISO(6OBA)_2_ (Midori Kagaku Co. Ltd.), adjusting the mixture concentrations to obtain the desired BP lattice orientation on each case following the method developed on our previous study^28^. ISO(6OBA)_2_: BP#1 (5.5%), BP#2 (5.2%), BP#3 (5.7%). All PDs dopants (MCF526, MCF528, MCF613 and MCF619) were prepared in chloroform solution and blended into each BP mixture at a 1 wt% concentration.

### Monocrystalline BP crystal preparation

Glass cells consisted of two indium-tin-oxide (ITO) coated ultraflat glass substrates assembled into a 10 µm thick sandwich cell. The ITO coated glass substrates were covered with nylon 6 polyamide, thermally conditioned, and rubbed in antiparallel configuration. Then, BP mixtures were filled into the cells by capillarity in isotropic phase. Polyamide nylon 6, as well as nylon 6–6, produced excellent monocrystalline and oriented BP crystals.

### BP phase transition analysis

BP precursor mixtures underwent a full thermal cycle to analyze the BP phase sequence while cooling down from isotropic state to cholesteric liquid crystal in an Instec HCS402 hot stage platform and STC 20U thermal controller and when illuminated either by a 365 nm LED spot lamp and a 550 nm LED source with intensity regulation for isomerization control light source. BP phases were analyzed by POM in reflection mode in an Olympus BX51 with a 5×/0.15 objective. Kossel patterns were obtained in the conoscopic configuration of the microscope using a 60×/0.70 objective with 550 nm/450 nm/400 nm light sources. BP reflection spectra were measured with an ocean optics flame-T spectrometer.

Additionally, we devised a system that allowed us to obtain the real images of the BP textures while briefly being illuminated by a white light source without affecting the PDs conformation, that would help with fast spectra acquisition, as well. At the lowest lighting power of our system, a shutter worked with a shutter speed of 1/500 s, illuminating the sample while acquiring an image at the same time. As a result, images of the BP textures showing their real reflection colors were obtained, as seen in the POM images shown in Figs. 1 and 3.

Response times were measured by electrooptical means. The obtained reflection signals upon photoisomerization were analyzed and recorded with a Tektronix TDS 2014 digital oscilloscope and a Tektronix OpenChoice acquisition software.

### Kossel analysis

Kossel patterns were simulated by modeling a body-centered cubic (BCC) or simple cubic (SC) crystal with a Kossel–Kikuchi pattern generator software modified for monocrystalline BPI BCC and BPII oriented SC structure, with different lattice constants and adapted for converging monochromatic lights of 450 and 400 nm. Orthographic projections of the Kossel lines corresponding to each pole were taken to obtain patterns fitting the experimental results and the lattice size for each BP crystal was estimated. The refractive index of the BP was assumed to be 1.6.

### Supplementary Information

Supplementary Information.

## Data Availability

All data generated or analyzed during this study are included in this published article and its supplementary information files.
