# Supplementary material for: All-optical 3D blue phase photonic crystal switch with photosensitive dopants
Source: Sci Rep. 2024 Apr 30;14:9910. doi: 10.1038/s41598-024-60667-6 (PMC11061285; doi:10.1038/s41598-024-60667-6)
Supplement: Supplementary file 1 — Supplementary Information. [file 41598_2024_60667_MOESM1_ESM.pdf]

SUPPLEMENTARY INFORMATION

All-Optical 3D Blue Phase Photonic Crystal Switch with Photosensitive Dopants

Eva Oton[1*,] Martin Cigl[2], Przemysław Morawiak[1], Sergei Mironov[2], Alexej Bubnov[2], Wiktor Piecek[1]

[1]Faculty of New Technologies and Chemistry, Military University of Technology, Warsaw, Poland

[2]Institute of Physics, Czech Academy of Sciences, Prague, Czech Republic

Corresponding author: eva.oton@wat.edu.pl

General synthetic experimental

All starting materials and reagents were used as purchased. All solvents used for the synthesis were "p.a." grade and used as received. ^{1}H NMR spectra were recorded on Varian VNMRS 300 instrument; deuteriochloroform ($CDCl_3$) and hexadeuteriodimethyl sulfoxide (DMSO-d_6) were used as solvents and signals of the solvent served as internal standard. Chemical shifts (δ) are given in ppm and *J* values are given in Hz. Signals were identified by APT, COSY and HMBC experiments. Elemental analyses were carried out on Elementar vario EL III instrument. The purity of all final compounds was checked by HPLC analysis (high-pressure pump ECOM Alpha; column WATREX Biospher Si 100, 250 × 4 mm, 5 μm; detector WATREX UVD 250) and were found to be >99.8 %. Column chromatography was carried out using Merck Kieselgel 60 (60−100 μm).

Synthetic procedures

*(E)-1-(4-Ethoxy-2,6-dimethylphenyl)-2-[4-(hexyloxy)phenyl]diazene (**MCF526**)*

Bromoethane (1.93 g, 12.30 mmol) was added to the stirred mixture of **1a** (3.30 g, 10.11 mmol) and anhydrous K_2CO_3 (1.40 g, 10.11 mmol) in DMF (40 mL) and the reaction mixture was heated to 45 °C. After ca. 6 h of stirring under anhydrous conditions, the resulting mixture was poured into water. The precipitate was filtered off, washed with water and pressed thoroughly. After drying under vacuum, the crude product was crystallised from ethanol (3×). Yield 2.69 g (75 %) as red crystalline solid. ^{1}H NMR ($CDCl_3$): 7.83 (2H, d, J = 8.8 Hz), 6.99 (2H, d, J = 8.8 Hz), 6.64 (2H, s), 3.90 - 4.19 (4H, m), 2.42 (6H, s), 1.73 - 1.89 (2H, m), 1.23 - 1.55 (9H, m), 0.92 (3H, t, J = 6.5). ^{13}C{^{1}H} NMR ($CDCl_3$): 161.12 (s), 158.38 (s), 147.28 (s), 144.69 (s), 133.94 (s), 123.94 (s), 114.85 (s), 114.57 (s), 68.32 (s), 63.41 (s), 31.56 (s), 29.16 (s), 25.69 (s), 22.61 (s), 19.97 (s), 14.85 (s), 14.05 (s). Elemental analysis: for $C_{22}H_{30}N_2O_2$ (354.49): calcd C 74.54, H 8.53, N 7.90, found C 76.46, H 8.51, N 7.89 %.

Ethyl (E)-4-((4-amino-2,6-dimethylphenyl)diazenyl)benzoate (3)

A solution of ethyl 4-aminobenzoate (**2**) (16.50 g; 0.10 mol) in a mixture of conc. H_2SO_4 (14 mL) and water (100 mL) was diazotized with a solution of $NaNO_2$ (10 g, 0.14 mol) in water (20 mL) keeping the temperature below 5 °C. The excess of nitrous acid was decomposed using a small amount of sulfamic acid. Formed diazonium salt solution was carefully neutralized using NaOH (10 g, 50%) at low temperature (≤ 5 °C) and then it was slowly added to the vigorously stirred solution of 3,5-dimethylaniline (15.30 g, 0.10 mol) in distilled water (195 mL) acidified with H_2SO_4 (5.6 mL) and crushed ice (ca. 150 g). The reaction temperature was kept below 10 °C. A red-brown precipitate forms rapidly during the addition of diazonium salt. Formed pale orange precipitate was filtered off, washed with cold water and pressed thoroughly to remove water. Filter cake was crystallized from ethanol. Yield 28.60 g (95 %). ^{1}H NMR (DMSO-d$_6$): 8.06 (2 H, d, *J*=8.7 Hz), 7.76 (2 H, d, *J*=8.7 Hz), 6.36 (2 H, s), 6.36 (2 H, br.s.), 4.32 (2 H, q, *J*=6.9 Hz), 2.48 (6 H, s), 1.34 (3 H, t, *J*=6.9 Hz).

Ethyl (E)-4-((4-cyano-2,6-dimethylphenyl)diazenyl)benzoate (4)

A fine powder of **3** (28.50 g, 0.10 mol) was suspended in the mixture of conc. H_2SO_4 (14 mL) and water (100 mL). After cooling to -5 °C, it was slowly (ca. 2 h) diazotized by powdered $NaNO_2$ (10 g, 0.14 mol) added in small portions, not to exceed 0 °C. The excess of nitrous acid was decomposed using a small amount of sulfamic acid. Formed diazonium salt was carefully neutralized using NaOH (10 g, 50%) at low temperature (≤ 5 °C). Neutralized diazonium was added portion-wise to the stirred CuCN (10.91 g, 0.12 mol) dissolved in the solution of KCN (16.0 g, 0.25 mol) in water (100 mL). Addition of the diazonium was accompanied by the formation of foam. The resulting mixture was let to settle overnight and then filtered. Filter cake was boiled with acetone (500 mL) for 30 minutes and then filtered again. Filtrate was evaporated and the solid crystallized from acetone. Yield 27.0 g (91 %). ^{1}H NMR ($CDCl_3$): 8.23 (2 H, d, *J*=8.8 Hz), 7.93 (2 H, d, *J*=8.8 Hz), 7.44 (2 H, s), 4.43 (2 H, q, *J*=6.7 Hz), 2.33 (6 H, s), 1.43 (3 H, t, *J*=6.7 Hz).

*(E)-4-((4-Cyano-2,6-dimethylphenyl)diazenyl)benzoate (**MCF528**)*

Solution of ester **4** (1.30 g, 4.65 mmol) in DMSO (10 mL) was added dropwise with stirring to the aqueous KOH (40 mL, 25%) heated to 50 °C. The mixture was stirred at this temperature until a clear solution was formed (ca. 30 min). Then it was poured into ice-water mixture and neutralized with conc. HCl. The formed precipitate was filtered off, washed several times with

water and dried in vacuum oven at 40 °C. Dried acid was dissolved in DMSO (10 mL), finely powdered K_2CO_3 (1.35 g, 9.77 mmol) was added, with stirring, followed by the addition of *n*-iodohexane (1.25 g, 5.78 mmol). The reaction mixture was stirred in a sealed reaction flask at 50 °C for 6 h. Resulting mixture was poured into water (40 mL) and extracted with diethylether (3 × 20 mL). Collected organic layers were washed with water and brine and dried over anhydrous $MgSO_4$. After evaporation of the solvent, the crude product was purified by column chromatography on silica using CH_2Cl_2 – acetone (99 : 1) as eluent. Yield after crystallization from ethanol: 1.25 g (75 %). ^{1}H NMR ($CDCl_3$): 8.22 (2 H, d, *J*=8.8 Hz), 7.93 (2 H, d, *J*=8.2 Hz), 7.43 (2 H, s), 4.37 (2 H, t, *J*=6.7 Hz), 2.32 (6 H, s), 1.70 - 1.92 (2 H, m), 1.22 - 1.57 (6 H, m), 0.91 (3 H, m). ^{13}C{^{1}H} NMR ($CDCl_3$): 165.78 (s), 154.60 (s), 154.22 (s), 133.20 (s), 132.62 (s), 131.51 (s), 130.66 (s), 122.48 (s), 118.61 (s), 111.58 (s), 65.56 (s), 31.40 (s), 28.61 (s), 25.65 (s), 22.51 (s), 18.36 (s), 13.97 (s). Elemental analysis: for $C_{22}H_{25}N_3O_2$ (363.19): calcd C 72.70, H 6.93, N 11.56, found C 73.31, H 7.01, N 11.65 %.

*(E)-3,5-difluoro-4-{[4-(hexyloxy)phenyl]diazenyl}phenol (**1b**)*

A fine suspension of 4-(hexyloxy)phenylammonium hydrogensulfate (17.7 g, 0.10 mmol) in concentrated acetic acid (50 mL) was diazotized with solid $NaNO_2$ (8.0 g, 0.11 mol) with stirring. The temperature of the reaction mixture was kept at about 10 ºC using an ice-water cooling bath. The resulting mixture was stirred for another 30 min while colling it to -10 °C. Then a small amount of urea was added, and the reaction mixture stirred well. The formed diazonium slurry was added portionwise to a solution of 3,5-diflurophenol (13.10 g, 0.10 mol) in a concentrated aqueous solution of NaOH (100 mL, 40%). The temperature of the reaction mixture was kept below -5 °C using an ice-water cooling bath, and the pH of the reaction mixture was kept in the basic region by occasional additions of Na_2CO_3 (ca 60.0 g in total). After the last addition of the diazonium salt solution, the reaction mixture was stirred thoroughly, and the separated precipitate was filtered off. Filter cake was washed it with water, diluted hydrochloric acid (1:5) and again with water. Yielded after crystallization from ethanol: 26.90 g (80 %). ^{1}H NMR (CDCl3): 7.87 (2 H, d, J = 8.8), 6.98 (2 H, d, J = 8.8), 6.52 (2 H, d, J = 10.0), 4.03 (2 H, t, J = 6.6), 1.81 (2 H, q, J = 7.1), 1.18 –1.57 (6 H, m), 0.88 (3 H, t, J = 6.5).

*(E)-3,5-Difluoro-4-{[4-(hexyloxy)phenyl]diazenyl}phenyl 4-butoxybenzoate (**MCF613**)*

A mixture of 4-butoxybenzoic acid (**6**, 1.01 g, 5.20 mmol) and azophenol **1b** (1.70 g, 4.96 mol) was dissolved in dichloromethane (25 mL). *N,N'*-dicyclohexylcarbodiimide (DCC, 1.18 g 5.55

mmol) and 4-(N,N-dimethylamino)pyridine (0.30 g, 2.46 mmol) were added and the mixture was stirred for 8 h at room temperature. Precipitated dicyclohexylurea was filtered off and the filtrate was diluted with dichloromethane (25 mL) and washed with HCl (20 mL, 1 : 15) and water. Organic layer was dried with anhydrous Na_2SO_4. Removal of the solvent under reduced pressure yielded crude product which was purified by column chromatography on silica with CH_2Cl_2-acetone (99 : 1) as eluent. Crystallization from hexane yielded 2.12 g (84 %) of **MCF613** as orange crystalline solid. ^{1}H NMR (CDCl3): 8.12 (2 H, d, *J*=8.8 Hz), 7.93 (2 H, d, *J*=8.8 Hz), 6.88 - 7.13 (6 H, m), 4.0 - 4.10 (4 H, m), 1.70 - 1.95 (4 H, m), 1.24 - 1.61 (8 H, m), 0.86 - 1.09 (6 H, m). ^{13}C{^{1}H} NMR ($CDCl_3$): 163.95 (s), 163.80 (s), 162.49 (s), 155.91 (dd, J = 259.0, 6.76), 151.12 (t, J = 14.0), 147.59 (s), 132.42 (s), 129.21 (t, J = 10.63), 124.99 (s), 120.42 (s), 114.68 (s), 114.43 (s), 106.90 (dd, J = 24.63, 3.38), 68.40 (s), 68.04 (s), 31.53 (s), 31.06 (s), 29.09 (s), 25.64 (s), 22.55 (s), 19.14 (s), 13.99 (s), 13.76 (s). Elemental analysis: for $C_{29}H_{32}F_2N_2O_4$ (510.58): calcd C 68.22, H 6.32, N 5.49, found C 67.97, H 5.46, N 11.65 %.

*(E)-4-{[4-(Hexyloxy)phenyl]diazenyl}-3,5-dimethylphenyl 4-butoxybenzoate (**MCF619**)*

Synthesis of this compound was analogous to the preparation of compound **MCF613**. Reaction of azophenol **Ia** (2.60 g, 7.96 mmol) with 4-butoxybenzoic acid (1.55 g, 7.98 mmol) in CH_2Cl_2 (50 mL) in the presence of dicyclohexylcarbodiimide (1.80 g, 8.46 mmol) and DMAP (0.30 g, 2.44 mmol) yielded 3.47 g (87 %). Chromatographic purification was performed in CH_2Cl_2-acetone 99.9 : 0.1. ^{1}H NMR (CDCl3): 8.15 (2 H, d, *J*=8.8 Hz), 7.89 (2 H, d, *J*=9.2 Hz), 6.90 - 7.09 (6 H, m), 3.96 - 4.20 (4 H, m), 2.38 (6 H, s), 1.74 - 1.98 (4 H, m), 1.27 - 1.64 (8 H, m), 1.0 (3 H, t, J = 6.7), 0.93 (3 H, t, J = 6.5). ^{13}C{^{1}H} NMR ($CDCl_3$): 164.99 (s), 163.51 (s), 161.73 (s), 149.87 (s), 148.97 (s), 147.04 (s), 132.50 (s), 132.25 (s), 124.30 (s), 121.99 (s), 121.58 (s), 114.65 (s), 114.26 (s), 68.39 (s), 67.98 (s), 31.56 (s), 31.14 (s), 29.14 (s), 25.68 (s), 22.59 (s), 19.18 (s), 19.11 (s), 14.02 (s), 13.81 (s).

Mesomorphic behavior of new photosensitive dopants

The mesomorphic properties of the photosensitive dopants were established by: (i) polarizing optical microscopy, POM, (NIKON ECLIPSE E600POL) coupled to a LINKAM LTS E350 heating/cooling stage and equipped with TMS 93 temperature programmer for temperature control, which enabled temperature stabilization within ± 0.1 K; and (ii) differential scanning calorimetry, DSC, (Perkin Elmer 8000). The measurements were done on samples of ~5-8 mg hermetically sealed in an aluminum pan on cooling/heating runs (with rate of 10 K min^{-1}) in a

nitrogen atmosphere. The temperature was calibrated on extrapolated onsets of melting points of water, indium and zinc.

On the basis of the POM and DSC results, the mesomorphic behavior for four new photosensitive dopants is summarized in Table S1. The microphotographs of the characteristic textures obtained on planar 12 μm thick samples are presented on Fig. S1. The second heating/cooling DSC runs for all photosensitive dopants are presented on Fig. S2. Materials with two phenyl rings on the molecular core, namely the MCF526 and MCF528 do not possess any liquid crystalline phase; only the isotropic-crystal (ISO-CR) phase transition was detected. Photosensitive dopants possessing three phenyl rings in the molecular core, namely MCF613 and MCF619, possess a broad and stable nematic (N) phase, ~130 K and 80 K, respectively. The POM and DSC fully confirm these results.

Table S1. The phases and phase transition temperatures of new photosensitive dopants. Melting point, m.p., and clearing point, c.p., in °C obtained on heating; phase transition temperatures, T_{tr}, and the temperature of crystallization, T_{cr}, on cooling. The corresponding enthalpy changes, ΔH, in Jg^{-1}, detected on the second cooling run at a rate of 10 $Kmin^{-1}$ are shown in square brackets.

**Compound**	**m.p.**	**c.p.**	**phase**	T_{cr}	**phase**	T_{tr}	**phase**
**MCF526**	73 [+103.0]	73 [+103.0]	**CR**	61 [-100.5]	**-**		**ISO**
**MCF528**	85 [+63.8]	85 [+63.8]	**CR**	50 [-75.0]	**-**		**ISO**
**MCF613**	90 [+86.0]	209 [+4.2]	**CR**	68 [-65.7]	**N**	208 [-4.4]	**ISO**
**MCF619**	95 [+77.4]	168 [+4.6]	**CR**	85 [-75.3]	**N**	166 [-4.6]	**ISO**

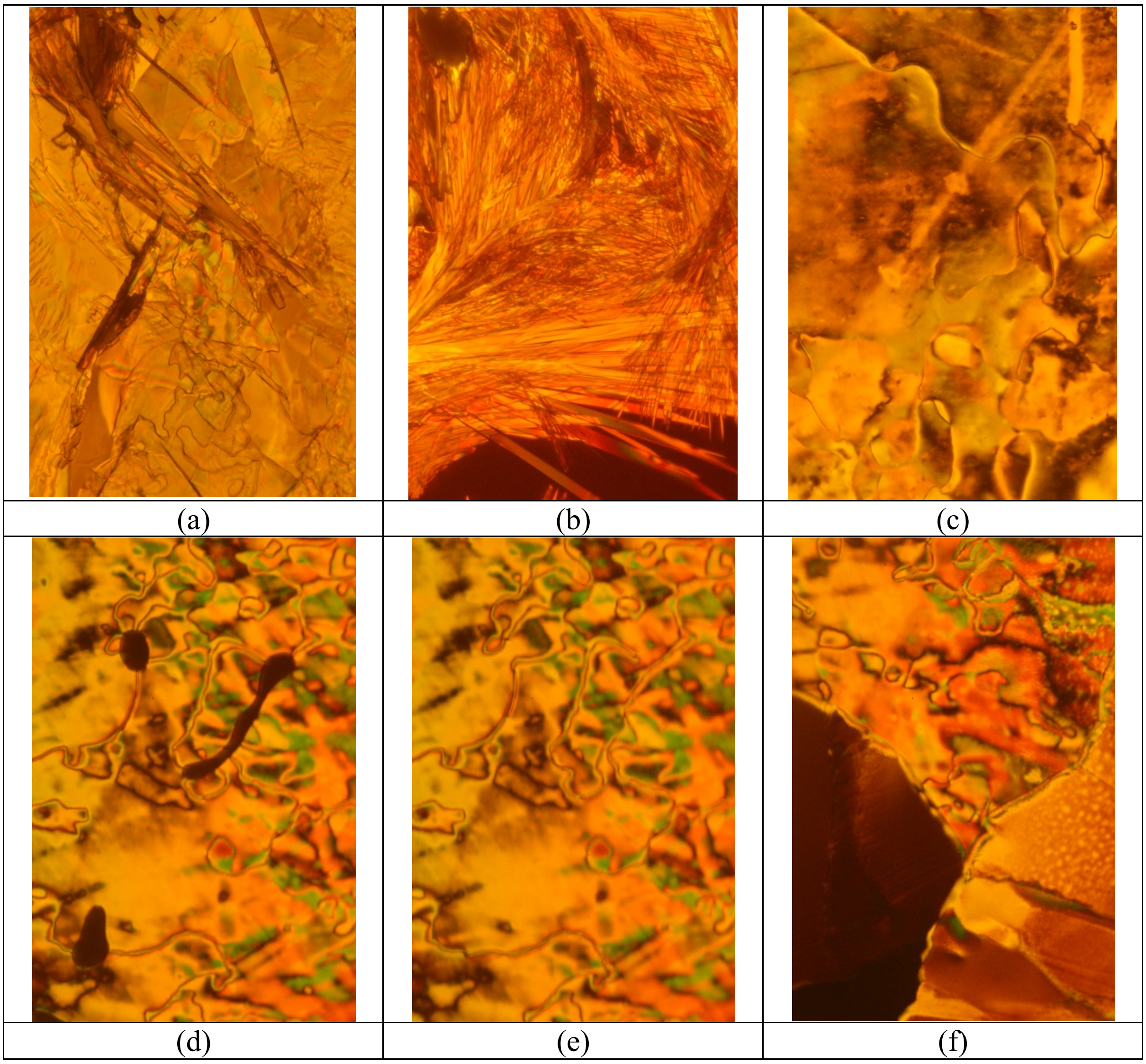

Figure S1. Photos of characteristic textures obtained on cooling for new photosensitive dopants: (a) the crystal phase for MCF526 compound at ~50°C; (b) the isotropic – crystal phase transition for MCF528 compound at ~52°C; (c) the Schlieren texture of the nematic phase for MCF613 compound at ~180°C; (d) the isotropic – nematic phase transition for MCF619 compound at ~165°C; (e) the Schlieren texture of the nematic phase for MCF619 compound at ~160°C; (f) the nematic – crystal phase transition for MCF619 compound at ~85°C. The width of all the microphotographs is~200 μm.

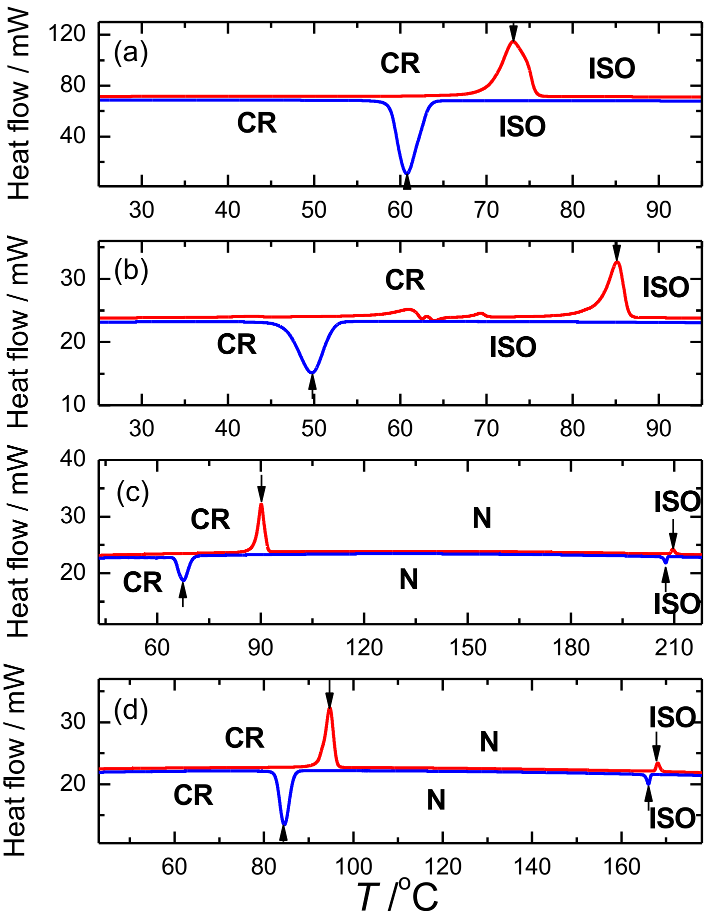

Figure S2. The second heating (red) and cooling (blue) DSC runs (10 Kmin^{-1}) for photosensitive dopants: (a) MCF526, (b) MCF528, (c) MCF613 and (d) MCF619. Vertical arrows indicate the phase transitions; mesophases are indicated.

UV-Vis spectral data for PDs

Table S2: UV-Vis absorption maxima and corresponding absorption coefficients of PDs measured in 1,2-dichloroethane.

Material	*E isomer*				*PSS@365 nm*			
	UV		Vis		UV		Vis	
	λ_{max} [nm]	ε [M^{-1}cm^{-1}]	λ_{max} [nm]	ε [M^{-1}cm^{-1}]	λ_{max} [nm]	ε [M^{-1}cm^{-1}]	λ_{max} [nm]	ε [M^{-1}cm^{-1}]
MCF526	358	23,270	448	1,994	356	7,201	451	2,079
MCF528	317	19,383	466	905	269	15,702	443	1,051
MCF613	338	24,734	449	1,400	311	14,333	436	1,732
MCF619	352	29,507	438	2,710	323	9,479	431	2,790

Table S3: UV-Vis absorption coefficients of PDs at used illumination wavelengths, measured in 1,2-dichloroethane.

	λ [nm]	*E isomer*	*PSS@365 nm*
		ε [$M^{-1}cm^{-1}$]	ε [$M^{-1}cm^{-1}$]
MCF526	365	21,596	6,679
	400	3,781	2,758
	550	28	245
MCF528	365	4,164	1,327
	400	311	450
	550	298	87
MCF613	365	25,138	3,880
	400	3,569	1,925
	550	2	26
MCF619	365	14,853	3,684
	400	1,291	1,046
	550	41	30
